# Imaging of the Acute and Chronic Cardiovascular Complications of Radiation Therapy

**DOI:** 10.1161/CIRCIMAGING.124.017454

**Published:** 2025-02-17

**Authors:** James Wilson, Chong Jun Hua, Nikoo Aziminia, Charlotte Manisty

**Affiliations:** 1 Barts Heart Centre, St Bartholomew’s Hospital, Barts Health National Health Service (NHS) Trust, London, United Kingdom (J.W., N.A., C.M.).; 2 Institute of Cardiovascular Science, University College London, United Kingdom (J.W., N.A., C.M.).; 3 Cardiology Department, National Heart Centre Singapore & Cardiovascular Sciences Academic Clinical Programme at Duke-National University of Singapore Medical School & Lee Kong Chian School of Medicine, Nanyang Technological University (C.J.H.).

**Keywords:** cardioonclogy coronary artery disease, echocardiography, heart failure, magnetic resonance imaging, radiation therapy

## Abstract

Chest radiotherapy (XRT) plays a crucial role in the treatment of a multitude of cancers including breast, lung, esophageal, and lymphoma. Although XRT enhances cancer survival rates, it may also expose healthy bystander tissues to radiation, potentially leading to severe complications. Initially considered relatively resistant to radiation damage, the heart has been shown over the past 4 decades to be susceptible to radiation-induced cardiovascular toxicity and despite advances in XRT which can minimize radiation exposure to heart tissue, no cardiac radiation dose is entirely safe. The clinical spectrum of radiation-induced cardiovascular toxicity is broad, encompassing coronary artery disease, myocardial dysfunction, valvular abnormalities, and pericardial disorders. Radiation-induced cardiovascular toxicity may manifest acutely or many years after XRT, with each condition more likely to present at certain time points post-XRT. Cardiac imaging is a crucial tool in both the screening and diagnosis of radiation-induced cardiovascular toxicity with an understanding of its pathophysiology, incidence, and progression required to implement a comprehensive, multimodality imaging approach to detect and manage these complications effectively.

Chest radiotherapy (XRT) is a critical treatment adjunct in many cancers. Although radiotherapy can improve survival, irradiation of bystander healthy cardiovascular tissue can result in complications, termed radiation-induced cardiovascular toxicity (RICT). RICT can occur both acutely and many years after completion of treatment, with certain manifestations more prevalent at certain time points. Imaging is key in the diagnosis and management of RICT, forming the bedrock of current international cardio-oncology guidelines. An understanding of the manifestations of RICT, the utility of a multimodality imaging approach, and emerging imaging tools enables physicians to manage these patients optimally.

## Pathophysiology and Manifestations of RICT

**Figure 1.**
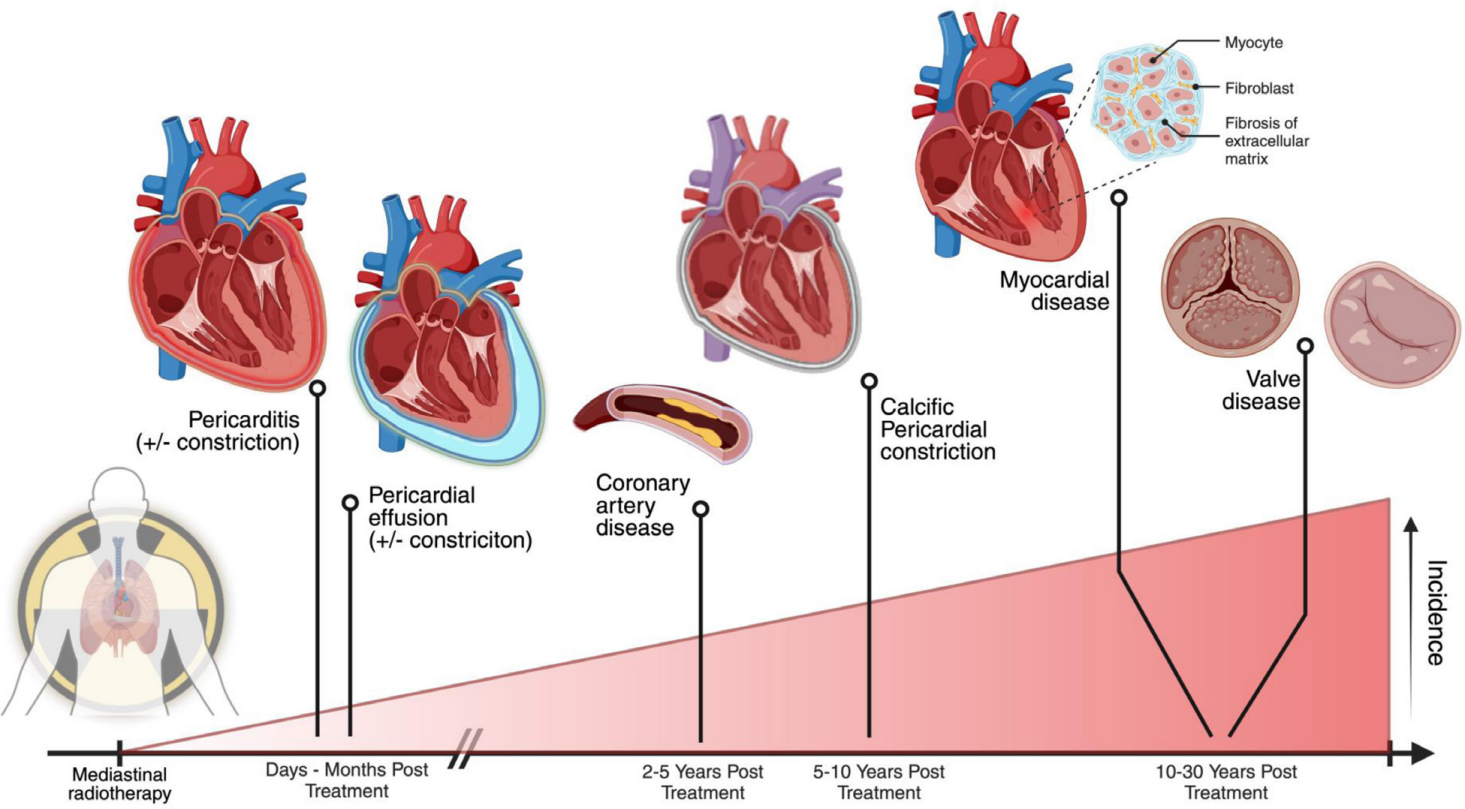
**Central illustration: manifestations of radiation-induced cardiovascular toxicity over time.** Created in BioRender. https://BioRender.com/n00z870.

Radiotherapy causes cardiovascular damage through oxidative stress, inflammation, endothelial cell proliferation, and fibrosis.^1,2^ Over time, this can manifest in different ways, with clinical manifestations presenting throughout treatment and late into survivorship (Figure 1: central illustration).

Acutely, XRT can cause pericarditis and effusions through inflammation and microvascular damage.^3^ Although relatively common historically, newer XRT techniques have rendered these pericardial complications less prevalent.^4^ Acute pericarditis and pericardial effusions may be asymptomatic and are typically self-limiting; however, they may rarely cause acute constriction and, if persistent, can lead to pericardial thickening, calcification, and years later, chronic constriction.^3^ Pericardial effusions may also develop due to damage to the pericardial venous and lymphatic systems.^3^ These slow-onset effusions are typically asymptomatic and picked up incidentally;^1^ however they can potentiate constriction and thus require monitoring. Tamponade secondary to radiotherapy-induced pericardial effusion is rare but documented.^3^

In coronary arteries, XRT can cause endothelial dysfunction and atheroma formation. The risk of coronary artery disease (CAD) increases 4- to 6-fold after XRT with a linear relationship to radiation dose,^5,6^ with a prevalence for the right and left anterior descending coronary arteries due to their anatomic orientation typically exposing them more to XRT fields.^7^ Asymptomatic CAD can develop as early as 2 years post-XRT, and clinical manifestations (such as angina, acute coronary syndromes, or ischemic heart failure) can occur as soon as 5 years post-XRT, especially in those with risk factors.^8^ A prospective study of 179 patients with Hodgkin lymphoma undergoing XRT between 2007 and 2012 found CAD in 34% of patients at 10 years post-treatment, at an average age of 42 years.^9^ Some have hypothesized that radiotherapy-induced CAD progresses quicker and requires monitoring even in asymptomatic survivors. Robust evidence to date is, however, limited to one study where computed tomography (CT) coronary artery calcium, used as a surrogate for CAD, increased more rapidly with higher radiotherapy doses.^10^

In the myocardium, radiation causes fibrosis leading to systolic and diastolic impairment.^11^ Patients typically develop clinical heart failure several years after XRT, although asymptomatic changes can occur as early as 6 weeks post-treatment.^12^ Heart failure with preserved ejection fraction—often with restrictive physiology—is >5× more common than heart failure with reduced ejection fraction.^13^

**Figure 2.**
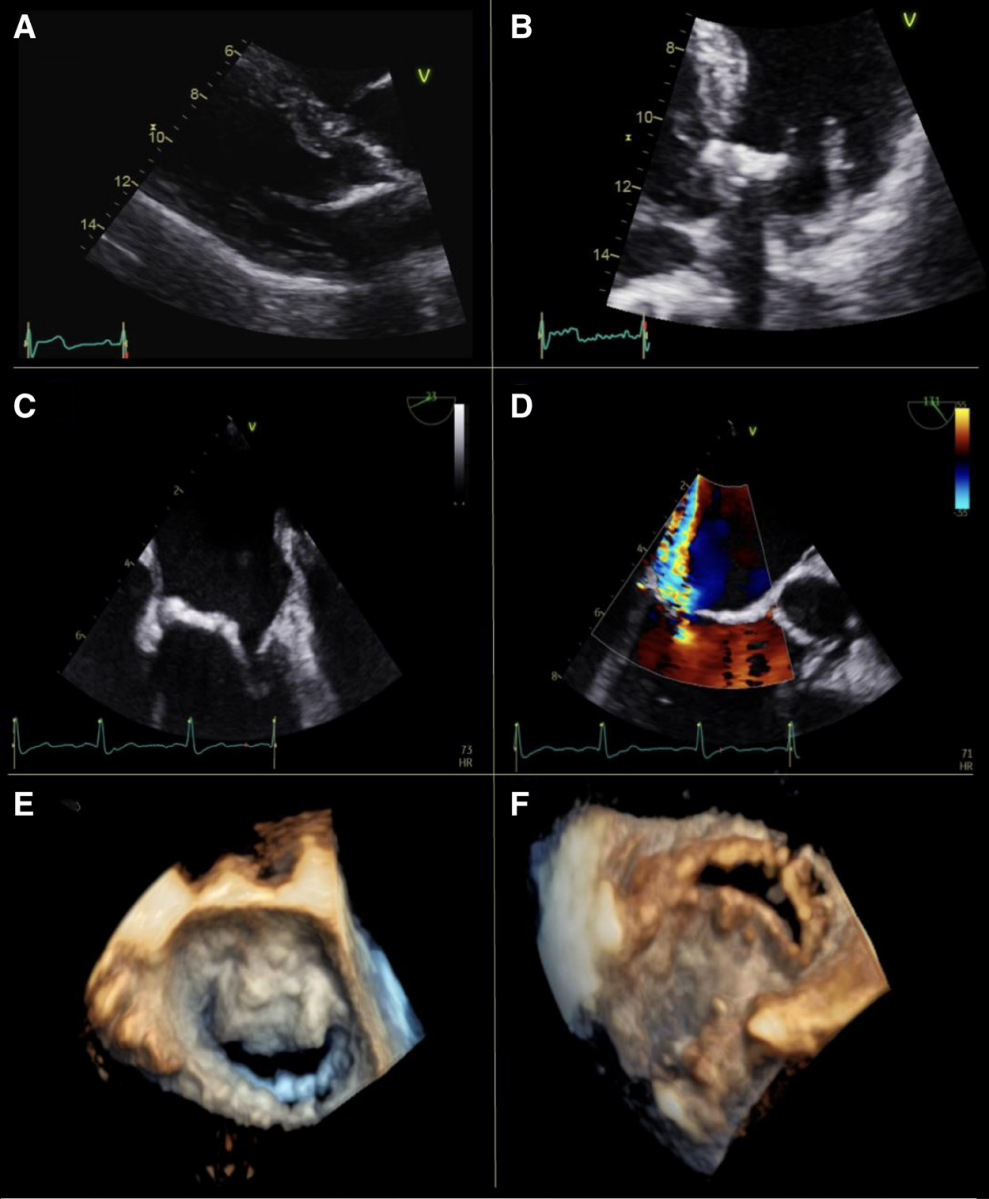
**Radiotherapy-induced mitral valve changes in a 60-year-old female treated with mantle field radiotherapy for lymphoma aged 24 years.** Transthoracic and transesophageal echocardiographic images **A** through **C**, Marked thickening and calcification of the basal anterior mitral valve leaflet and aorto-mitral curtain (**D**): posterior jet of mitral regurgitation (**E** and **F**): 4-dimensional models of the mitral valve, demonstrating relative sparing of the leaflet tips and commissures. **E** represents surgical view and **F** represents view from left ventricle.

Radiation-induced fibrosis combined with hemodynamic loading leads to valve leaflet and subvalvular apparatus damage, predominantly of the mitral and aortic valves.^1,14^ Typically sparing the valve tips and commissures, thickening, fibrosis, and calcification of the annulus, basal, and mid leaflets are hallmarks (Figure 2).^15^ Valve disease normally develops slowly; incidence of moderate or greater valvular disease in 415 patients who underwent XRT for Hodgkin lymphoma was 1% at 10 years and 9% at 25 years.^16^

## Risk Factors for RICT

The main risk factor for the development of RICT is cardiac irradiation dose, with a 4% to 11% relative risk increase of major adverse cardiac events per Gray of cardiac irradiation^8^ and a linear risk relationship for the development of CAD.^5^

Age at time of XRT is equally an important factor (younger age at exposure means increased cumulative risk over a lifetime), as are the traditional cardiovascular risk factors such as diabetes, smoking, and preexisting cardiovascular disease.^17^ Across over 2000 patients with breast cancer who had undergone XRT between 1958 and 2001, the presence of any cardiovascular risk factor doubled the probability of major adverse cardiac events, with previous ischemic heart disease conveying an over 6-fold risk.^5^

One emerging area of interest is the impact of irradiation of specific cardiac structures on the risk of RICT.^18^ High radiotherapy doses to the left anterior descending coronary artery are an independent predictor of major adverse cardiac events and all-cause mortality,^19^ and these vulnerable cardiac structures receive the greatest irradiation in lung and esophageal cancers.^20^

## Imaging for Cardiovascular Screening in Asymptomatic Patients Peri-XRT

Expert consensus guidelines recommend screening for asymptomatic cardiac disease pre-XRT to improve cardiotoxicity risk assessment and post-XRT to enable early detection and management of RICT.^21,22^ Despite a lack of large-scale prospective trial data, these guidelines are in broad agreement in terms of imaging modality and frequency, with slight variation between societies.

### Pretreatment Assessment

As part of baseline cardiotoxicity risk stratification before radiotherapy, both the European Society of Cardiology guidelines and International Cardio-Oncology Society expert consensus statement recommend considering echocardiography in higher-risk patients with previous cardiovascular disease or with cardiovascular symptoms. Cardiovascular magnetic resonance imaging (CMR) should be utilised when echocardiography is not available or is non-diagnostic.^22^ Review of prior thoracic CT images (cancer diagnostic or staging scans) for coronary calcium as a surrogate for CAD is also recommended to improve cardiovascular risk stratification and guide the administration of primary preventative medications.^21^

### Post-Radiotherapy

**Figure 3.**
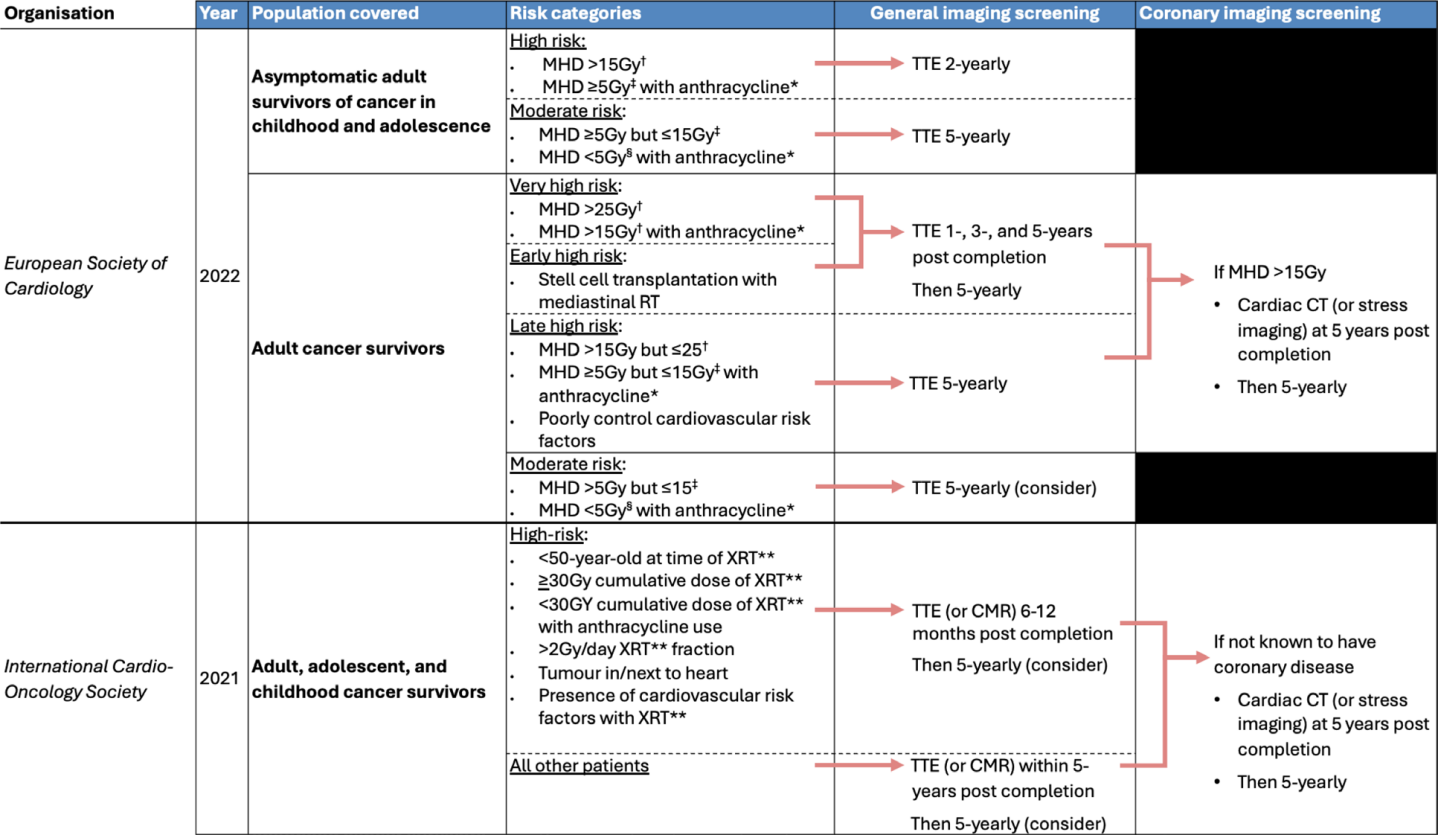
**European Society of Cardiology and International Cardio-Oncology Society image screening guidelines post–chest radiotherapy (XRT).** CMR indicates cardiovascular magnetic resonance imaging; CT, computed tomography; Gy, Gray; and TTE, transthoracic echocardiogram. †If mean heart dose (MHD) is not available, prescribed radiotherapy dose (RT) ≥35 Gy to a volume exposing the heart. ‡If MHD is not available, prescribed RT 15–34 Gy to a volume exposing the heart. §If MHD is not available RT <15 Gy to a volume exposing the heart. *At dose of/equivalent to ≥100 mg/m^2^ of doxorubicin. **With heart in treatment field.

After XRT, the timing and modality of imaging selected will depend on the patient’s baseline cardiovascular risk, XRT exposure (mean heart dose, MHD), and the use of any concomitant cardiotoxic systemic anticancer therapy.^21,22^ The European Society of Cardiology recommends echocardiography at 12 months after treatment in patients at high risk, with subsequent echocardiography at 3 years, 5 years, and then 5 yearly. In lower-risk patients, echocardiography every 5 years is recommended (Figure 3). Although the International Cardio-Oncology Society recommends surveillance for asymptomatic CAD at 5 years post-treatment in all patients, with serial imaging every 5 years, the European Society of Cardiology guidelines limit this to patients treated with MHD >15 Gy. The imaging modality chosen for assessing CAD should be guided by local expertise and availability. These recommendations are based on historical expert consensus statements and retrospective cohort data,^18^ with no prospective studies validating serial screening for CAD in asymptomatic patients after XRT. Similarly, no studies have trialed empirical standard or intensive primary prevention of CAD in the post-XRT population.

### Imaging for Cardiovascular Intervention Planning Post-XRT

After XRT, patients largely have worse outcomes for cardiovascular interventions, including for both surgical and transcatheter aortic valve procedures.^23^ Coronary stenting and angioplasty had been associated historically with increased mortality and restenosis rates, although more recent evidence suggests this is not the case.^24^ Surgery may be complicated by XRT-induced internal mammary artery disease, gross calcification of the great veins or aorta (porcelain aorta), and radiotherapy-induced pulmonary fibrosis.^25^ Guidelines, therefore, advise preoperative CT angiography to assess internal mammary artery patency and quality and aortic calcification before bypass grafting.^21,22^

## Imaging of Acute Cardiovascular Complications of Radiation Therapy

Acutely, the most common cardiovascular complications of XRT are pericarditis and pericardial effusions. For symptomatic patients presenting during (or immediately after) radiotherapy, transthoracic echocardiography is therefore used first line given its low-risk profile, wide availability, and ability to assess the pericardium in detail alongside any hemodynamic effects. Additional multimodality imaging is commonly required to aid diagnosis and plan subsequent management.^17^ Acute pericardial complications may also be detected on routine surveillance cancer CT imaging.

### Acute Pericarditis and Pericardial Effusions

**Figure 4.**
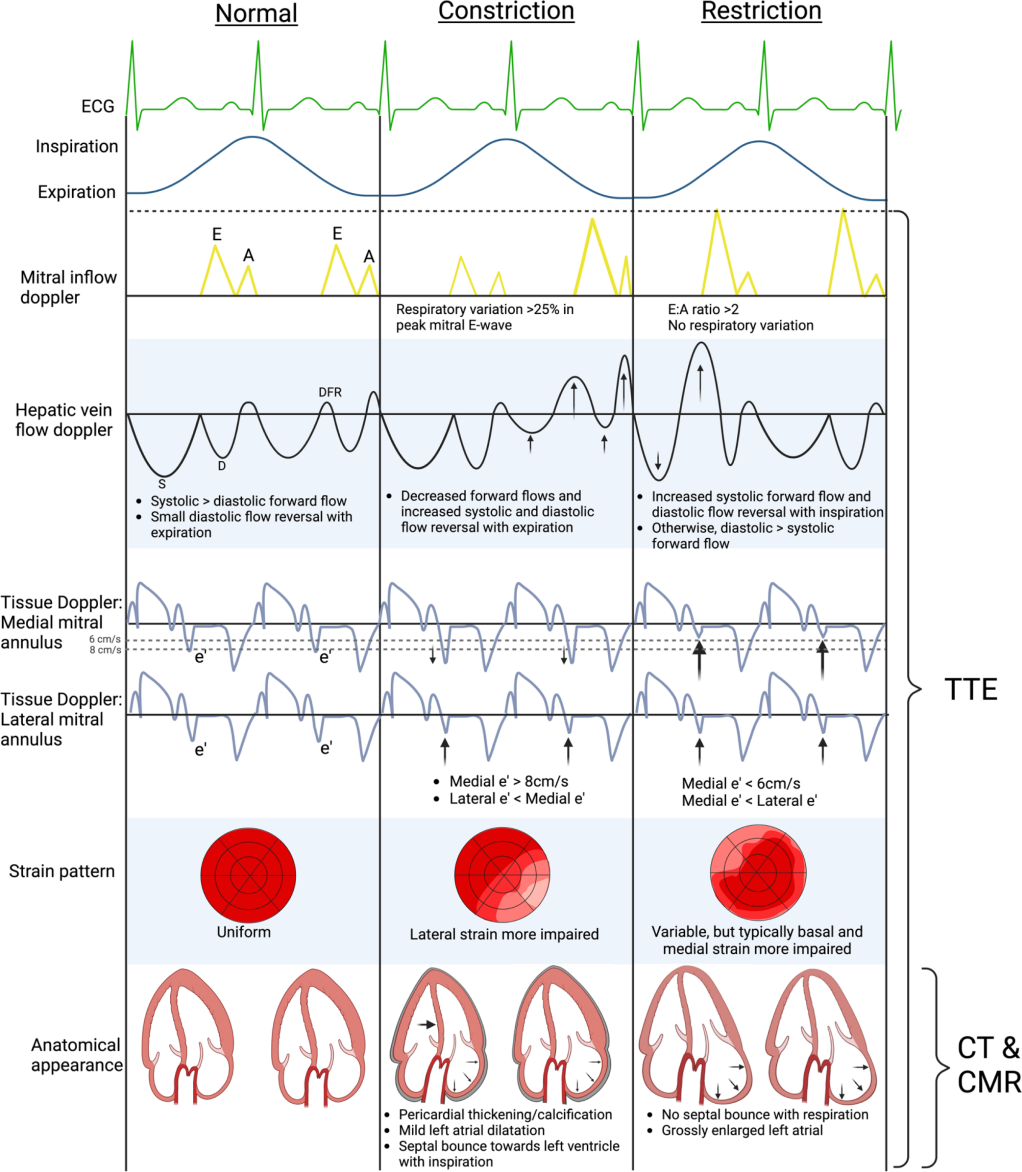
**Multimodality differentiation between constrictive pericarditis and restrictive cardiomyopathy.** A indicates mitral A-wave inflow; CMR, cardiovascular magnetic resonance imaging; CT, computed tomography; D, diastolic forward flow; DFR, diastolic flow reversal; E, mitral E-wave inflow; e′, mitral e′ value; S, systolic forward flow; and TTE, transthoracic echocardiogram. Created in BioRender. https://BioRender.com/n00z870.

In isolated pericarditis, echocardiography generally shows a thickened and echogenic pericardium, with multiple parallel reflections seen on M-mode imaging.^26^ Rarely, patients may develop acute constrictive physiology, and therefore, full echocardiographic assessment including diastolic physiology and respiratory variation should be performed. Indicators of constrictive physiology (summarized in Figure 4) include septal bounce (due to abrupt cessation of diastolic filling in a noncompliant pericardium), exaggerated respiratory variation of mitral and tricuspid valve Doppler inflows (>25% and >40%, respectively), annulus reversus (reversal of the usually higher lateral compared with medial mitral annulus peak tissue Doppler velocities), and increased hepatic diastolic flow reversal with expiration.^26^

Echocardiography may also identify pericardial effusions, while simultaneously evaluating for hemodynamic effects (ie, tamponade), and guiding management and intervention. Pericardial effusions appear as an echo-free space, darker than pericardial fat, and external to the myocardium but anterior to the descending aorta. Although tamponade is a clinical diagnosis, echocardiographic features include diastolic dysfunction, dilated (noncollapsing) inferior vena cava (IVC), exaggerated respiratory variation of mitral and tricuspid valve E-wave inflows (>30% and >60%, respectively), and chamber collapse.^26^ Right atrial chamber inversion duration of >1/3 of the cardiac cycle has 100% specificity and 94% sensitivity of 94% for tamponade, with right ventricular (RV) diastolic inversion having lower accuracy.^26^ The pattern of impaired diastolic ventricular filling also differs between tamponade and constrictive pericarditis, with filling impaired throughout diastole in the former and predominantly early in diastole in the latter.^26^

**Figure 5.**
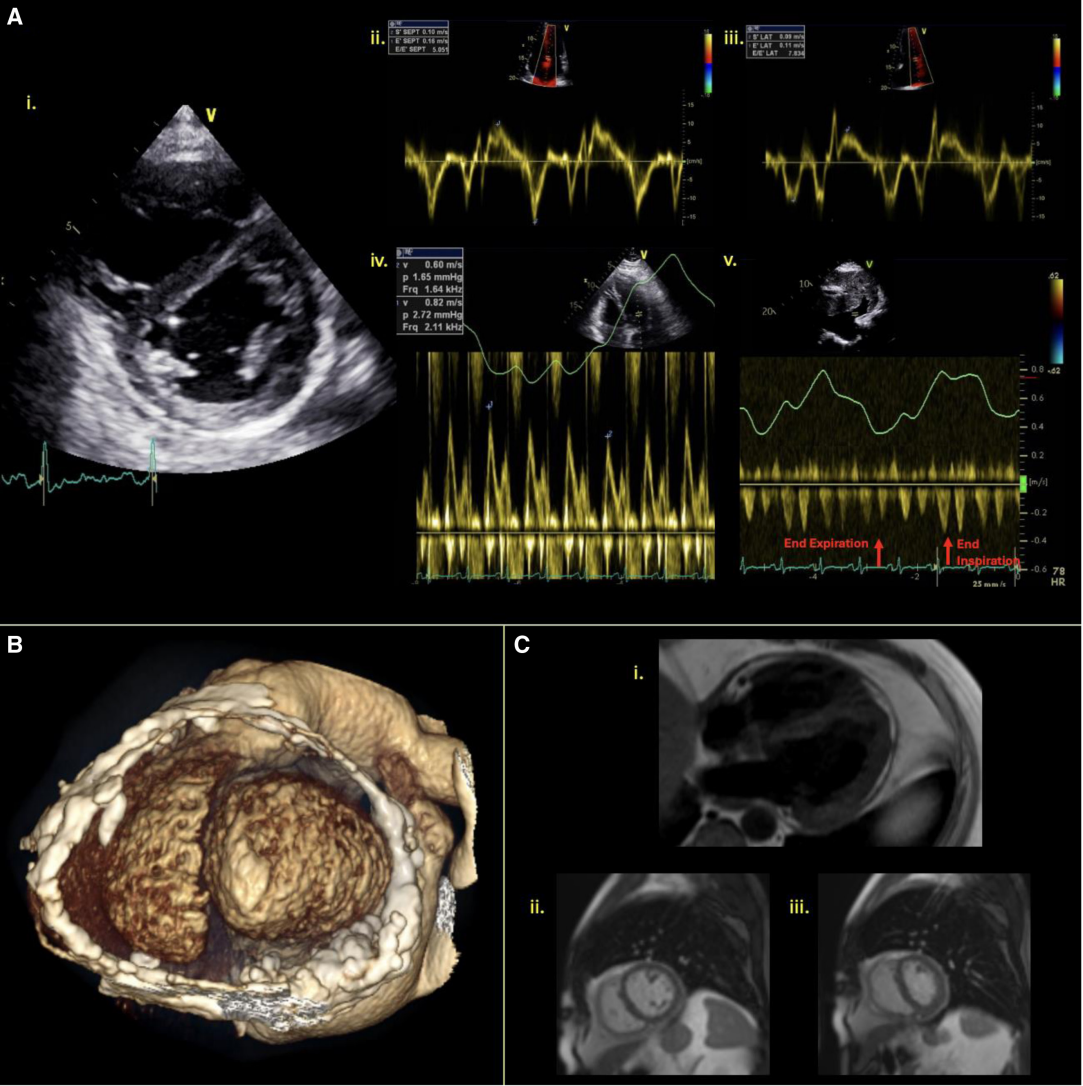
**A 54-year-old man with pericardial constriction after chest radiotherapy for lymphoma aged 18 years. A**, Transthoracic echocardiography images demonstrating pericardial thickening, increased echogenicity as well as septal flattening with inspiration (**i**), annulus reversus with e′ sept 0.16 m/s and e′ lateral 0.11 m/s (**ii** and **iii**) >25% respiratory variation of transmitral E-wave velocity (**iv**) and decreased hepatic vein forward flow with expiration (**v**). **B**, Three-dimensional computed tomography reconstruction showing extensive pericardial calcification. **C**, Cardiovascular magnetic resonance imaging showing pericardial thickening on T1 black-blood imaging (**i**) and deformation of left ventricular with inspiration (**ii**) with resolution with expiration (**iii**).

CMR has greater diagnostic sensitivity than echocardiography for identifying pericarditis, and so should be considered when clinical signs are suggestive but echocardiography is normal.^27^ CMR is also able to assess physiology and identify acute inflammation, protocols should be comprehensive in order to assess both the pericardial thickening, the physiological effects and perform tissue characterisation for identifying inflammation and calcification. Black-blood T1 weighted turbo spin echo imaging with the addition of a fat-saturation prepulse permits differentiation of pericardial fat from the thickened pericardium, allowing accurate pericardial measurement. T2-weighted imaging using the short-tau inversion-recovery sequence highlights areas of increased free water seen with inflammatory edema; hence, increased pericardial signal is seen with acute pericarditis. Late gadolinium enhancement (LGE) imaging will identify edema and fibrosis, common with acute pericarditis. Recently, a criterion for grading the severity of pericardial disease based on LGE has been proposed, although this has not been validated in the context of radiotherapy.^28^ Real-time gradient echo cine imaging during deep free breathing in the 4-chamber view and mid-short axis slices can assess for ventricular interdependence, with ventricular septal shift towards the LV on inspiration suggestive of constrictive physiology (Figure 5). Assessing for concomitant myocarditis with T2 short-tau inversion-recovery, T2 mapping, and LGE imaging should also be performed, with diagnosis made according to the Lake Louise Criteria.^29^

Cardiac CT with a dedicated protocol and ECG gating, cine imaging, and 4-dimensional multiplanar reconstruction volume-rendered imaging with contrast offers an alternative to CMR for the diagnosis of acute pericarditis and is a good discriminator of acute from chronic pericarditis; Calcification; Figure 5, suggests disease chronicity.^27^ The sensitivity and specificity of CT for diagnosis of pericardial effusions is slightly lower than for CMR,^3^ but hemodynamic consequences can also be determined by assessing for right atrial and RV collapse, alongside markers of RV overload, such as dilatation of the IVC, septal bounce on cine imaging, or venous reflux of contrast on CT.^26,27^

As well as allowing for the assessment of size, location, and any tamponade, both CT and CMR tissue characterization can aid discrimination between transudative and exudative pericardial effusions, reducing the need for diagnostic pericardiocentesis.^4^ On CT, a value of ≥4.7 Hounsfield units has 80% sensitivity and 87.7% specificity for exudative pericardial effusions.^30^ By CMR, T1 mapping values at 1.5T of ≥3015 ms suggest a transudate, with an area under the curve of 0.93 (95% sensitivity and 81% specificity).^31^

## Imaging of Chronic Cardiovascular Complications of Radiation Therapy

Cardiovascular complications of XRT more commonly present chronically, years or even decades, after treatment. Echocardiography is again recommended first line, but a multimodality approach is often required depending on the clinical presentation, local expertise, and treatment pathway.

### Coronary Artery Disease

Cardiac CT coronary angiography can identify coronary atheroma anatomically before the development of ischemia, enabling preventative management. Cardiac CT coronary angiography has a high negative predictive value for excluding CAD, and a lower radiation exposure (1–4 mSv) than invasive coronary angiography or myocardial perfusion scanning.^17^ CT coronary artery calcium scans, which solely look for calcified coronary lesions, have lower radiation exposure with no contrast requirement. Pre-XRT coronary artery calcium scoring has been shown to better predict future cardiovascular events than Framingham risk scores, while post-XRT, it has a high negative predictive value in excluding CAD.^4,32^ Newer multidetector CT scanners enable coronary artery calcium assessment from nongated images, with feasibility demonstrated on low-dose lung cancer screening scans.^33^ Given almost all oncology patients undergo thoracic CT imaging both before and after treatment, using these scans for cardiovascular risk stratification is attractive and has recently been used in cancer patients to refine risk models.^34^

Screening for subclinical coronary disease is currently recommended in the European Society of Cardiology guidelines, with surveillance imaging via cardiac CT coronary angiography 5 yearly after radiotherapy in patients who have received radiotherapy doses >15 Gy MHD, even if asymptomatic.^22^ The evidence to support this recommendation is limited, and in practice, implementation may be challenging. For childhood and young adult cancer survivors, the International Late Effects of Childhood Cancer Guideline Harmonization Group concludes that there is insufficient evidence to support primary screening.^35^

CAD can also be suspected from resting regional wall motion abnormalities on echocardiography, although post-XRT nonischemic RICT fibrosis can also cause regional myocardial hypokinesia.^36^ Routine surveillance echocardiography after radiotherapy is recommended for all childhood and adult cancer survivors excluding those at low risk, with the frequency determined by MHD and the dose of any anthracycline given.

**Figure 6.**
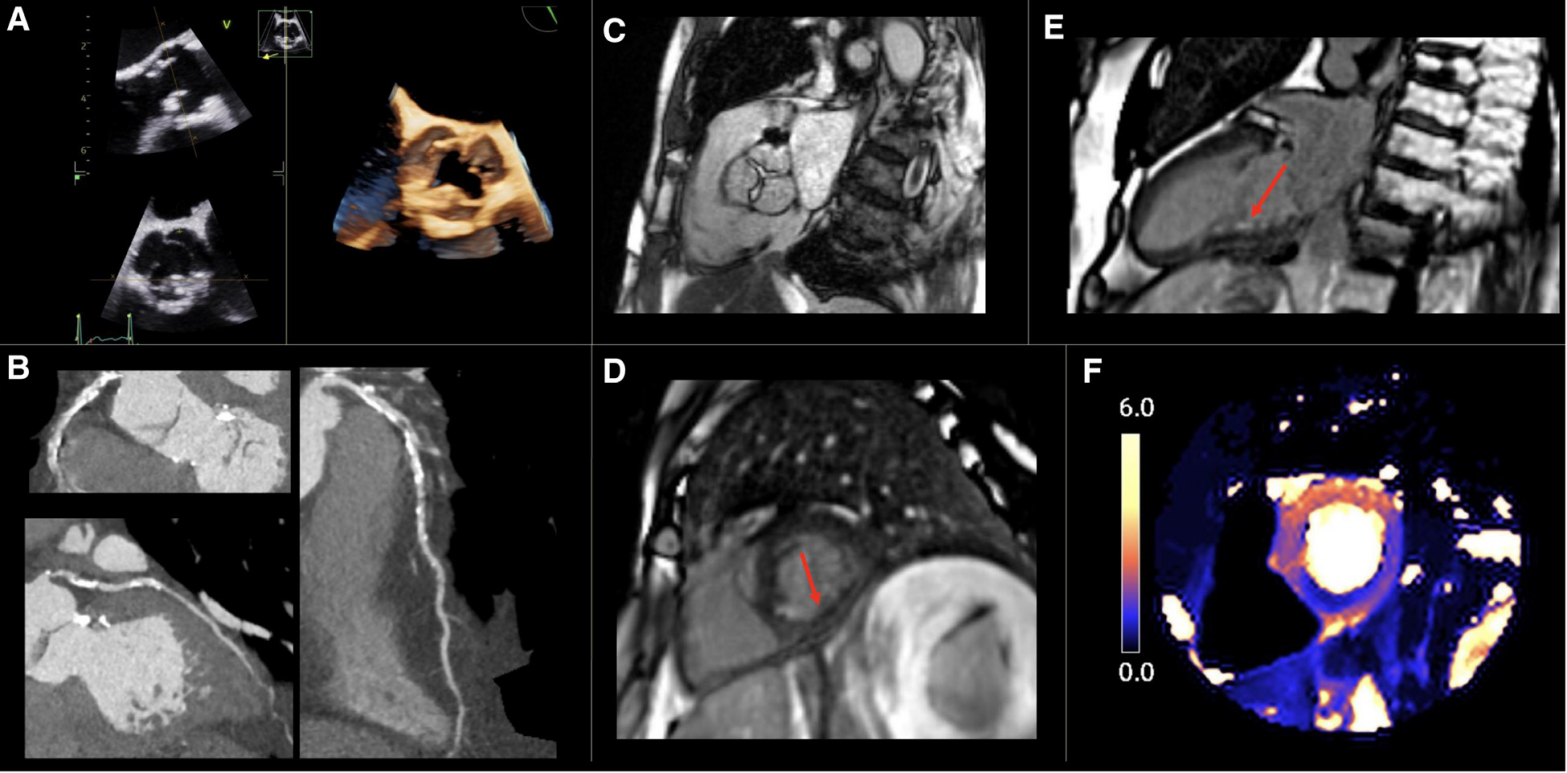
**Multimodality imaging demonstrating radiation-induced cardiovascular toxicity in a 72-year-old man who had mantle radiotherapy aged 38 years for Hodgkin lymphoma.** Patient had presented with exertional breathlessness and chest pain with transthoracic echocardiogram demonstrating aortic stenosis. Transesophageal echocardiography (**A**) demonstrated typical radiotherapy-induced changes of the aortic valve, while computed tomography coronary angiogram (**B**) showed diffuse coronary disease with severe disease in proximal left anterior decending, left circumflex and right coronary arteries. Patient went on to have cardiovascular magnetic resonance imaging which showed severe aortic stenosis through planimetry of the valve on cine imaging (**C**), late gadolinium enhancement in the basal-mid inferior walls (arrows) suggesting subendocardial infarction (**E** and **D**), and adenosine-induced reductions in stress myocardial blood flow in the basal inferior, inferolateral, and inferoseptal segments using quantitative perfusion mapping (**F**).

For patients with symptoms suggestive of ischemia, the choice of anatomic or functional imaging modalities should follow standard guidelines.^37^ Cardiac CT coronary angiography can identify prognostically important atheroma (Figure 6), and moderate lesions can be further evaluated for functional significance using CT fractional flow reserve, helping to guide subsequent intervention.^38^ Stress echocardiography has a high specificity (89%) and positive predictive value (87%), with a reasonable sensitivity (59%) for functionally significant CAD detection and prediction of future coronary events in both symptomatic and asymptomatic patients post-XRT.^2,17,36^

Nuclear myocardial perfusion scanning using single-photon emission computed tomography (SPECT) has, compared with stress echocardiography, a higher sensitivity (65%) but lower specificity seen (11%) for ischemia post-XRT.^36^ Perfusion defects at rest may be secondary to fibrosis and have doubtful clinical significance.^39^ Myocardial perfusion scanning with positron emission tomography (PET) provides several advantages over SPECT. It has higher spatial and temporal resolution, uses less radiation, is faster, and has a higher sensitivity and specificity with good performance in the post-XRT population.^40^ Tracers such as rubidium-82, [^15^O]H_2_O, [^13^N]ammonia, and the recently approved flurpiridaz, allow PET to give reliable quantification of absolute myocardial blood flow and myocardial flow reserve due to their more linear uptake than SPECT tracers, allowing for the assessment of both microvascular and diffuse epicardial coronary disease which SPECT is unable to do accurately.^40^ In the post-XRT population, both of these are important assessments to be made in patients presenting with chest pain, due to a higher likelihood of either significant ostial disease resulting in balanced ischemia or microvascular disease resulting in ischemic symptoms.

CMR meanwhile can assess CAD both indirectly, using cine imaging to assess for regional wall motion abnormalities and LGE to look for ischemic scar, and directly with stress myocardial perfusion imaging utilizing regadenoson or adenosine to induce stress (Figure 6). Although stress perfusion CMR has not been validated specifically after XRT, large post-XRT-studies have demonstrated a high prevalence of inducible perfusion defects using CMR in keeping with CAD.^41,42^ Until recently, stress perfusion CMR was largely qualitative, relying on visual identification of relative differences between myocardial segments and thus not being able to reliably identify multivessel and microvascular disease. New quantitative perfusion mapping techniques however can estimate myocardial blood flow on a pixel-by-pixel basis at stress and rest. Validated against coronary angiography and fractional flow reserve measurements, fully automated quantitative myocardial blood flow maps of high-resolution free-breathing perfusion sequences can be generated in-line on the scanner after image acquisition.^43^ This allows for both rapid and straightforward identification of both single vessel, multivessel, and microvascular myocardial ischemia as well as identification of ischemic scar in the post-XRT population using CMR.

### Valvular Disease

Transthoracic echocardiography should be used first line for RICT valvular disease, with severity graded following standard national and international guidelines.^44^ Three-dimensional imaging is particularly useful in the assessment of valvular commissures and leaflet tips (Figure 6); these typically are relatively spared compared with the basal leaflets and annulus (in contrast to other causes of valvular heart disease).^15^ Planimetry using transesophageal echocardiography may be required to accurately determine the severity of mitral valve disease. Transthoracic echocardiography–derived mitral valve area calculations in mitral stenosis can be distorted due to concomitant XRT-induced diastolic dysfunction shortening the pressure half time and elevating the mitral E-wave, while in mitral regurgitation annular calcification can distort transthoracic echocardiography annulus diameter measurement and calculation of the effective orifice area and regurgitant fraction. Transesophageal echocardiography is, however, higher risk post-XRT due to possible radiotherapy-induced esophageal injury.

**Figure 7.**
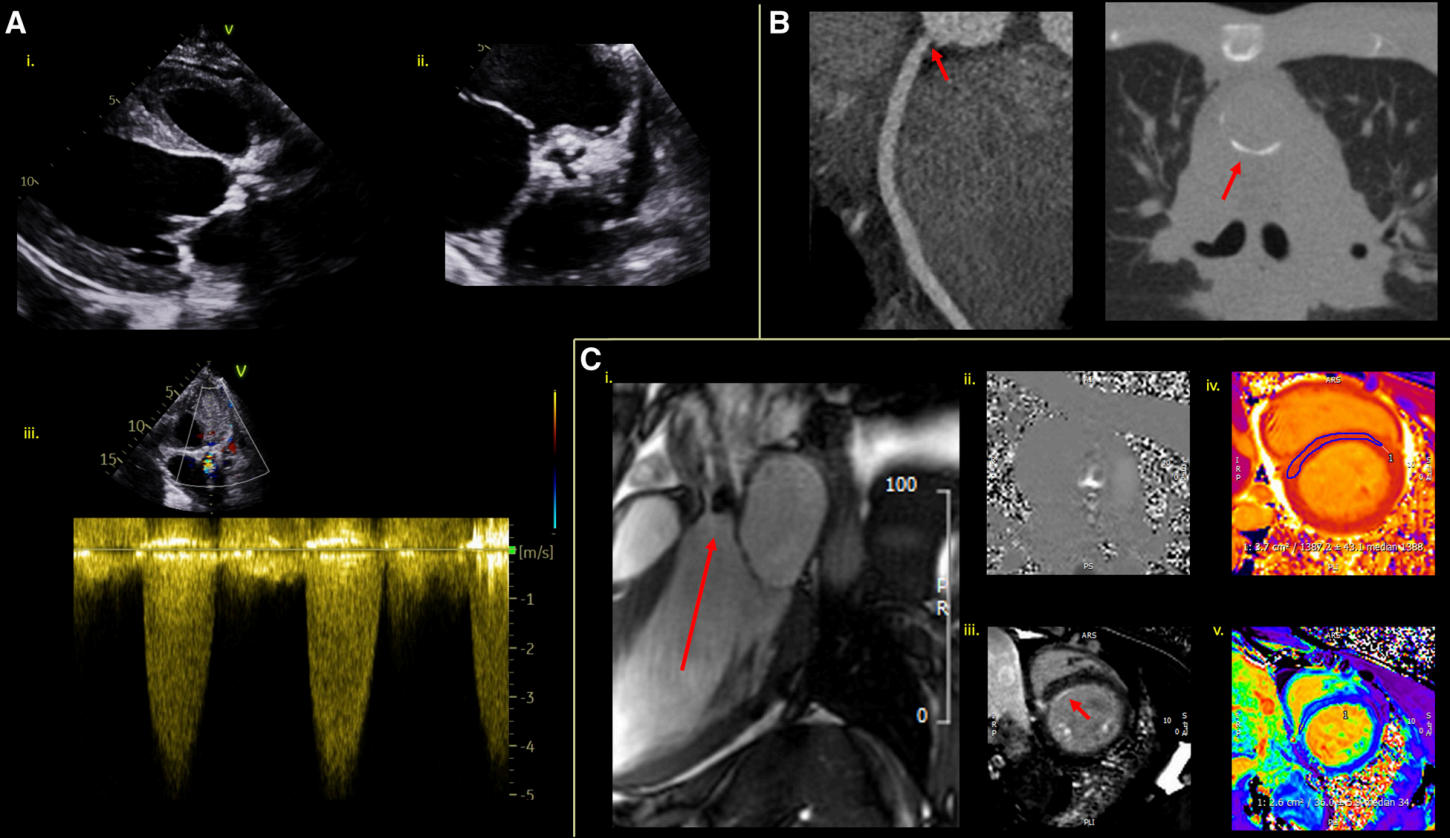
**A 29-year-old man who had undergone mantle field radiotherapy for lymphoma aged 4 years. A**, Transthoracic echocardiography image showing visually dilated left ventricle (**i**), heavy calcification of the aortic valve (**ii**), and severe aortic stenosis (Velocity-max of 4.57 m/s; **iii**). **B**, Cardiac computed tomography coronary angiography showing noncalcified ostial disease (**left**) as well as calcification of posterior aorta (**right**). **C**, Cardiovascular magnetic resonance imaging on 3T scanner showing marked aortic thickening and stenosis on 3-chamber cine (**i**) and flow mapping (**ii**), with tissue characterization showing areas of basal subendocardial and intramyocardial septal late gadolinium enhancement (**iii**, arrow), as well as raised septal T1 MOLLI at 1387 (normal values, 1220–1360) (**iv**) and raised septal extracellular volume (ECV) at 34% (**v**).

After echocardiographic assessment, CT provides invaluable information of the valvular and annular calcification, and surrounding cardiac and extra cardiac structures to guide intervention planning (Figure 7).^21,22^ It also shows high-resolution cross-sectional anatomic information of the valves and is well validated for planimetry of aortic stenosis and regurgitation.^45^

CMR meanwhile provides accurate measurement of ventricular volumes, alongside using phase-contrast velocity mapping to calculate peak velocities, and regurgitant volumes and fractions to enable evaluation of severity.^46^ Planimetry of the aortic valve using CMR can be performed accurately for aortic stenosis (Figures 6 and 7) and the regurgitant orifice measured in aortic regurgitation.^46^

### Left Ventricular Dysfunction

Echocardiographic measurement of left ventricular ejection fraction (LVEF) remains an important cornerstone in assessment of XRT-induced systolic dysfunction. Given the high inter and intraobserver variability of 2-dimensional biplane LVEF assessment, 3-dimensional echocardiography is recommended where feasible.^22^ LVEF assessment may also miss early myocardial dysfunction, hence speckle-tracking global longitudinal strain measurement is now recommended routinely, using a threshold for cardiotoxicity of >15% relative reduction from baseline.^22^ Regional strain may also add value, with segments with abnormal myocardial strain found to correlate with areas of XRT exposure, and impairment in global longitudinal strain to correlate with MHD.^47,48^

**Figure 8.**
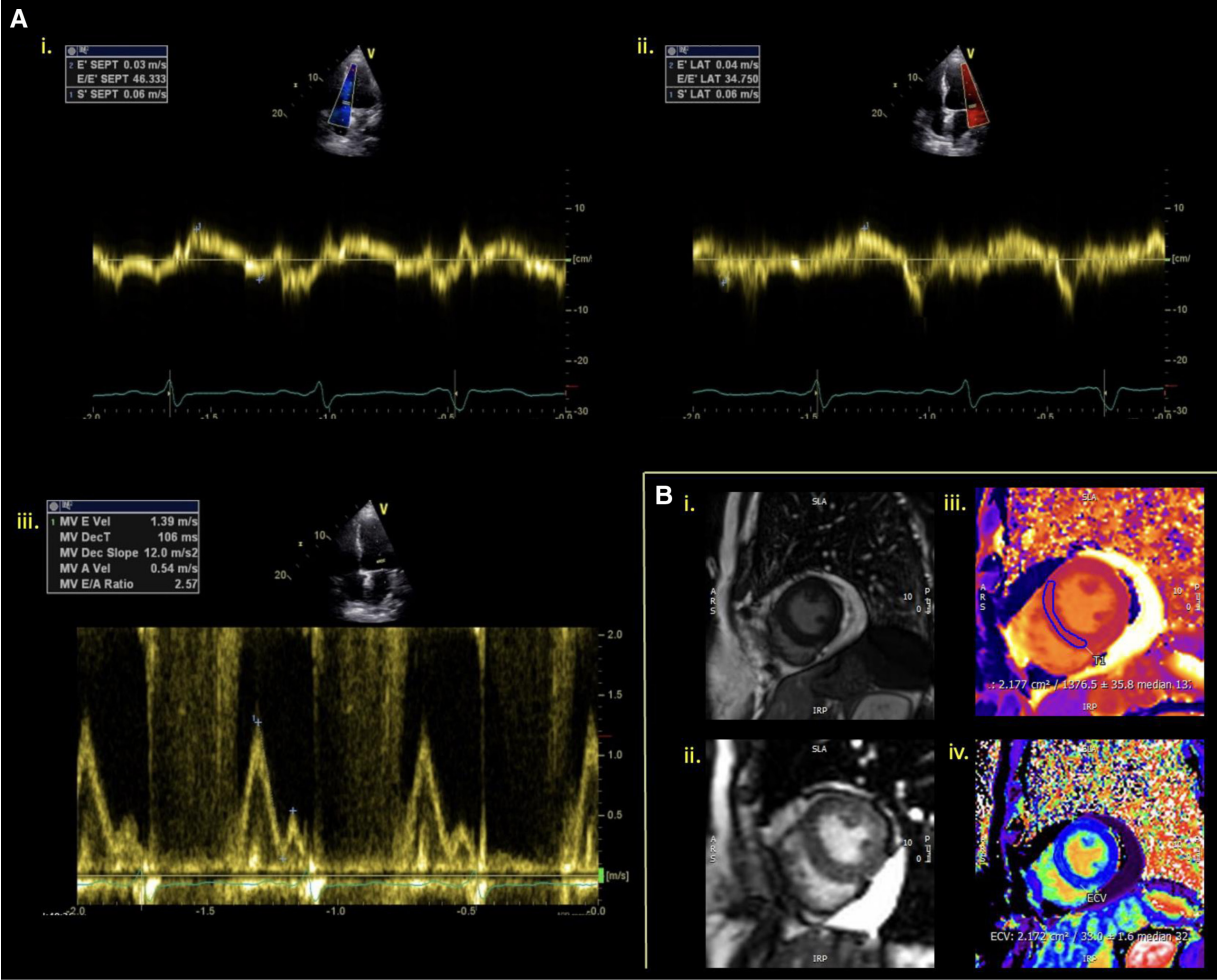
**Echocardiogram demonstrating restrictive physiology in a 53-year-old female patient who had undergone radiotherapy 16 years previously for lung cancer. A**, Transthoracic echocardiogram tissue Doppler of medial (**i**) and lateral (**ii**) mitral annuluses showing decreased e′ velocities. Pulsed wave Doppler of the mitral valve (MV) inflow showing E-wave>A-wave velocity (E Vel, A Vel) with a ratio of 2.57 with short deceleration time (DECT) of 106 ms (**iii**). Average E-wave/e′ of 40.54 and septal (SEPT) e′<lateral (LAT) E′ in keeping with restriction. **B**, Cardiovascular magnetic resonance imaging (CMR) (**i**) scout (**ii**) late gadolinium enhancement (LGE) imaging (**iii**) T1 MOLLI mapping and (**iv**) extracellular (ECV) mapping on 1.5T Scanner. Raised T1 at 1376 ms (normal values 970–1050 ms) and ECV at 32%.

Given the prevalence of restrictive cardiomyopathy post-radiotherapy, a comprehensive echocardiographic assessment of diastolic function is essential. Summarized in the Table and as seen in Figure 8, severe left atrial dilatation, mitral inflow E-wave/A-wave ratio >2 and decreased medial e′ velocities on tissue Doppler are all hallmarks of restrictive physiology.^49^

**Table.**
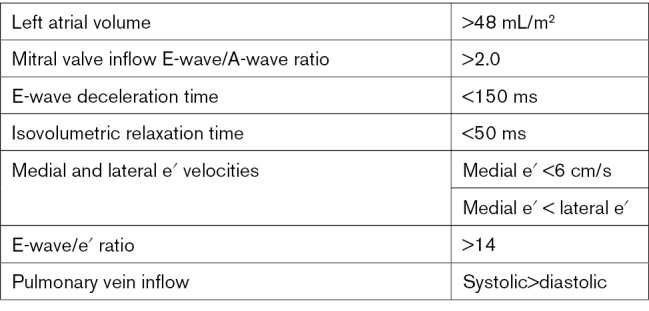
American Society of Echocardiography/European Association for Cardiovascular Imaging/Mayo Clinic Echocardiographic Criteria for Restrictive Physiology^49^

More recently, left atrial strain has been demonstrated to have greater sensitivity for detecting early diastolic dysfunction than other techniques.^50^ Left atrial strain has recently been incorporated into national guidelines for diastolic assessment, with reservoir strain <18% and pump strain <8% suggesting diastolic dysfunction.^51^

CMR may offer additional insights, particularly after abnormal echocardiography or where imaging is nondiagnostic (e.g., in patients with breast prostheses). CMR LVEF assessment is considered gold standard and in cancer patients has been shown to have a higher accuracy and precision than echocardiography.^52^ Myocardial strain can also be measured either with dedicated myocardial tagging and strain-encoded sequences or via postprocessing of standard cine images with feature tracking algorithms. CMR-derived strain has comparable accuracy to speckle-tracking echocardiographic strain,^53^ and its use has been demonstrated in the post-XRT population with impaired strain parameters in 80 asymptomatic lymphoma patients at 20 years post-XRT, when compared with matched controls.^54^

Myocardial tissue characterization using parametric mapping techniques (T1, exracellular volume) enables quantification of interstitial fibrosis and is commonly abnormal post-radiotherapy (Figures 7 and 8).^17^ LGE imaging can identify areas of focal fibrosis due to direct radiotherapy-induced myocyte injury, as well as rule out alternative causes of cardiac dysfunction such as XRT-related ischemic heart disease.^55^

Nuclear multigated acquisition imaging can assess both systolic and diastolic function, with diastolic dysfunction identified from prolongation of the isovolumetric relaxation phase and delayed ventricular filling time on ventricular time-activity curves.^40^ Multigated acquisition imaging however requires radiation and does not interrogate other cardiovascular structures, making it a less attractive imaging modality for assessing for chronic cardiovascular effects of radiotherapy.^40^ Equally, the use of gated cardiac CT for serial ventricular functional assessment is limited by its requirement for irradiation and contrast. However, there may be select groups, where imaging of the coronary arteries or pericardium is also required, where cardiac CT may be used for LV functional assessment.^4,21,22^

### Constrictive Pericarditis

Given that both constrictive pericarditis and restrictive cardiomyopathy are seen post-radiotherapy with similar presentations, differentiating between these can be diagnostically challenging.^21^ Echocardiographic criteria can help distinguish between the two conditions, although a multimodality managing approach is often required both for diagnosis and management planning (Figure 4). Despite imaging advances, cases may remain unclear due to coexistent disease clouding key echocardiographic parameters or early disease having not caused significant anatomic changes, in which case invasive hemodynamic assessment may be required.

Assessment for late-onset constrictive pericarditis should be assessed like acute pericarditis, but pericardial calcification is also common; hence, cardiac CT offers added value.^2^ CMR can detect both the structural and functional abnormalities seen in constrictive pericarditis while identifying features in keeping more with restrictive cardiomyopathy.^56^ LGE and T2-weighted imaging are less commonly abnormal in chronic constrictive pericarditis, where calcification and chronic fibrosis rather than active inflammation are more prominent pathological processes. Surgical planning should assess the extent and location of pericardial calcification from CT images, both to plan the regions of the pericardium to be stripped, and to assess for mediastinal fibrosis or aortic calcification that may complicate already high-risk pericardial surgery.^2^

## Future Developments and Evidence Gaps

Despite the advancements that have been made in the understanding of RICT and its imaging, several important aspects remain unanswered. Emerging imaging techniques are likely to impact in due course on our ability to diagnose, screen, and manage these conditions.

There is increasing awareness of the effect of XRT on RV function but to date a relative lack of research in this area. Long-term studies of RV function post-XRT using traditional echocardiographic measures (e.g., tricuspid annular plane systolic excursion) have confirmed a similar correlation with MHD as for LVEF.^57^ RV strain is rarely used clinically, though is well validated for early detection of XRT-induced RV dysfunction.^58^ Although CMR studies of RV assessment post-XRT are scarce, the improved measurement accuracy over echocardiography, alongside additional myocardial tissue insights may add incremental value.^59^

Advances in nuclear imaging may also be able to increase our ability to diagnose RICT early, through direct visualization of myocardial tissue damage. Novel tracers such as ^123^I-metaiodobenzylguanidine SPECT imaging and [^11^C]-meta-hydroxyephedrine CT-PET imaging visualize sympathomimetic denervation.^40^ ^111^In-antimyosin PET imaging, meanwhile, is a specific marker for myocardial cell injury and necrosis.^40^ Although neither of these techniques has been demonstrated thus far in radiotherapy-induced cardiac disease, they offer an interesting area of future development and research.

Current international guidelines recommend asymptomatic surveillance screening for both higher-risk adults and all childhood cancer survivors; however, evidence is needed to demonstrate impact on clinical outcomes and cost-effectiveness. There is recognition that modern radiotherapy techniques deliver XRT with much lower MHDs, therefore prevalence of RICT is likely to fall.^20^ Furthermore, new radiotherapy methods, such as proton beam therapy, convey significantly lower exposure of the heart to radiation ^60^ Surveillance guidance should be tailored to address this, with long-term studies required to inform these recommendations.

Although risk stratification using MHD dose is reflected in the guidelines, the dose to specific cardiac structures is not, despite evidence demonstrating impact on cardiovascular sequelae.^61^ Further knowledge from imaging studies (such as the CARdioimaging in Lung Cancer PatiEnts Undergoing Radical RadioTherapy (CARE-RT) study)^62^ of how the location of radiation deposition within the heart affects the risk of RICT will allow better personalization of XRT in the future, including creation of dose constraints to specific cardiac substructures when required.

Understanding the role and efficacy of cardioprotective agents (such as statins or colchicine) to prevent cardiovascular toxicity during or after XRT is also important, given promising results from preclinical models.^63^ Given the challenge and cost of assessing for chronic cardiotoxicity from acute cardioprotective studies, improved specific cardiac phenotyping via both blood and imaging biomarkers will be required as surrogate markers for the prediction of late effects.

## Conclusions

Cardiac imaging is vital both before, during, and after XRT for cardiotoxicity risk stratification, early diagnosis, and planning management of RICT. RICT can take many forms, with certain conditions including pericardial disease more likely to present acutely and others such as valve and coronary disease more prevalent years after XRT administration. A personalized multimodality imaging approach is required based on the likely cardiovascular pathology, with resources directed to those patients at the highest risk.

## Article Information

### Sources of Funding

Professor Manisty is supported directly and indirectly by the National Institute for Health and Care Research and Biomedical Research Centers at University College London Hospitals and Barts Health National Health Service Trusts.

### Disclosures

Professor Manisty has speaker and advisory roles for Pfizer, Beigene, Biotronik, and Abbott St Jude Medical and is a Co-Founder of MycardiumAI. The other authors report no conflicts.
